# Popularity of Surgical and Pharmacological Obesity Treatment Methods Searched by Google Users: the Retrospective Analysis of Google Trends Statistics in 2004–2022

**DOI:** 10.1007/s11695-023-06971-y

**Published:** 2023-12-16

**Authors:** Mikołaj Kamiński, Maja Miętkiewska-Dolecka, Matylda Kręgielska-Narożna, Paweł Bogdański

**Affiliations:** 1 Department of the Treatment of Obesity and Metabolic Disorders, and of Clinical Dietetics, Poznań University of Medical Sciences, Szamarzewskiego 84, 60-569 Poznań, Poland; 2 Student Scientific Club of Clinical Dietetics, Department of the Treatment of Obesity and Metabolic Disorders, and of Clinical Dietetics, Poznań University of Medical Sciences, Szamarzewskiego 84, 60-569 Poznań, Poland

**Keywords:** Google Trends, Obesity, Bariatric surgery, Dietary supplements, Infodemiology, Weight loss

## Abstract

**Purpose:**

Many individuals search for obesity treatment options on the Internet. We aimed to analyze the popularity of pharmacological and surgical obesity treatment methods searched by Google users.

**Material and Methods:**

We used Google Trends to identify topics representing the following: recommended surgical methods (*n* = 9), recommended pharmacological methods (*n* = 10), and not recommended pharmacological methods (*n* = 34). The data was generated for 2004–2022 and 2020–2022. Relative search volume (RSV) was adjusted using “Gastric bypass surgery” as a benchmark. We analyzed the geographical and temporal trends of the topics.

**Results:**

In 2004–2022, the topics representing recommended surgical methods numerically gained the most popularity among Google users, but in 2020–2022 the recommended drugs exceeded other obesity treatment methods. The most popular individual topics since 2004 were “flaxseed,” “Spirulina,” “Carnitine,” “Bariatric surgery,” and “Orlistat.” The most dynamic increases of searches since 2004 were observed for “Sleeve gastrectomy,” “Curcumin,” “Psyllium,” and “Bupropion/Naltrexon.” Since 2018, topics representing GLP-1 analogs such as “Semaglutide” and “Saxenda” revealed exponential increases in RSV, causing that “Semaglutide” to become the fourth most popular topic in 2020–2022.

**Conclusions:**

Google users across the world were the most interested in topics representing bariatric surgery, but recently recommended drugs for the treatment of obesity gained the most attention. The most popular individual topics were dietary supplements with uncertain effects on weight loss.

**Graphical Abstract:**

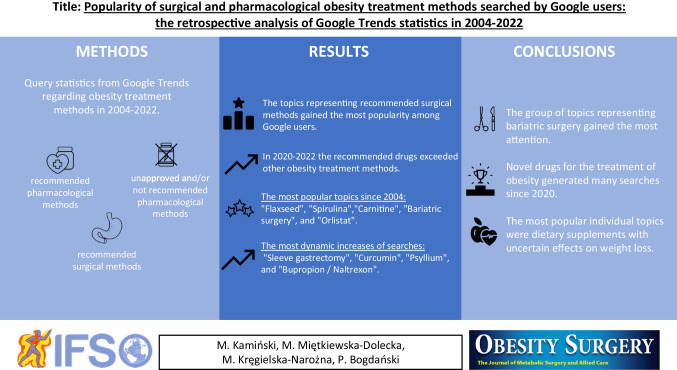

**Supplementary Information:**

The online version contains supplementary material available at 10.1007/s11695-023-06971-y.

## Introduction

Obesity is one of the most pressing global health concerns, which decreases both life expectancy and disability-adjusted life years [1]. Due to the multifactorial etiology of obesity, low compliance, and tendency to relapse, obesity is a challenging condition to treat. The standard of care in the management of obesity recognizes lifestyle modification as the primary intervention [2]. The next lines of treatment include pharmacotherapy, and bariatric surgery.

Newly approved anti-obesity agents can be expensive without insurance coverage. Moreover, bariatric surgery is associated with preoperational risk and further complications, which frighten some individuals with obesity [3]. Given these challenges, individuals with obesity often explore alternative treatment methods. Additionally, some people may turn to various medicaments and their combinations in pursuit of rapid weight loss. Indeed, several agents previously used to treat obesity have been banned because of severe adverse events [4, 5]. Simultaneously, there is a large, poorly regulated market of dietary supplements for weight loss with questionable efficacy in treating obesity [6].

The Internet has become a primary source for health-related information, with Google being the world’s most popular search engine [7]. Analyzing Google queries through Google Trends (GT) appears to be a viable tool for gaining insight into the interests of Google users globally within specific health domains, e.g., diets [8] and dietary supplements [9].

To date, no study compared the interest of Google users in different treatment methods for obesity. Previous papers using GT compared only bariatric surgery methods [10, 11] or GLP-1 analogs [12]. We hypothesize that analysis of GT may present trends of the popularity of different methods: current surges and past fads. Moreover, the analysis may reveal if not recommended methods have comparable or even higher popularity among Google users than recommended drugs and procedures.

In this paper, we aimed to analyze the popularity of recommended and not recommended pharmacological and surgical obesity treatment searched by Google users.

## Material and Methods

This is an infodemiological study utilizing freely available data provided by GT (https://trends.google.com/trends/); thus, the project did not require Ethical Committee approval. Our claim was confirmed by the Ethical Committee of our University (written communication; KB-1011/22; 7 December 2022). The study does not violate the Declaration of Helsinki 1964.

### Data Collection

GT presents Google query statistics. A user can write in an interesting search term and choose a specific region (worldwide or specific country) and period (since January 2004). GT presents a time trend representing a sample of 1% of all searches [13]. The results are expressed as relative search volume (RSV), which ranges between 0 and 100. 0 represents a complete lack of interest (0%), while 100 corresponds to the peak of popularity (100%) in the given period and location. Importantly, RSV is adjusted to the number of Google users in the chosen period and location [14]. GT users may write in up to five different terms at once, and GT will generate up to five time trends at once. In such circumstances, RSV is adjusted, and 100 will correspond to the highest popularity of one of the chosen phrases.

GT provides an option to recognize some inputs as “topic.” While search terms are the exact words written in GT, topics are proposed by the engine when it recognizes phrases related to the popular query. The topic may be matched by the same word but written in different languages. For example, the input “eagle” treated as a search term will be analyzed by GT literally; thus, RSV will be the highest in English-speaking regions. However, if the input “eagle” will be matched as the topic “Eagle,” it will include all queries associated with the topic in all available languages. For that reason, topics are more useful in studies utilizing GT than search terms [8, 9].

All details about selecting search terms are presented in Supplementary File 1.

We matched the following topics (*n* = 10) representing recommended pharmacological methods of obesity treatment: “Amfenaprone” (matched by typing “diethylpropion”), “Benzphetamine,” “Bupropion/Naltrexone,” “Liraglutide,” “Orlistat,” “Phentermine,” “Phentermine/Topiramate,” “Saxenda,” “Semaglutide,” and “Xenical.”

Further, we found the following topics representing unapproved and/or not recommended pharmacological (mostly dietary supplements) methods of obesity treeatment (*n* = 34) in the GT search engine: “β-Methylphenethylamine,” “1,3-Dimethylbutylamine,” “2,4-Dinitrophenol,” “Alpha Lipoic Acid,” “Beta-glucan,” “Bumetanide,” “Candyleaf,” “Caralluma,” “Carnitine,” “Cetilistat,” “Chitosan,” “Clenbuterol,” “Conjugated linoleic acid,” “Curcumin,” “Deterenol,” “Dexfenfluramine,” “Ephedra,” “Fenfluramine,” “Fenproporex,” “Flaxseed,” “Garcinia Cambogia,” “Glucomannan,” “Higenamine,” “Kalahari cactus” (matched hoodia gordonii), “Lorcaserin,” “Mangosteen,” “Octodrine,” “Oxilofrine,” “Phenylpropanolamine,” “Rimonabant,” “Sibutramine,” “Spirulina,” “Vachellia rigidula,” and “White kidney bean.”

Finally, the following topics (*n* = 9) representing recommended surgical methods were matched: “Adjustable gastric band,” “Bariatric surgery,” “biliopancreatic diversion,” “Duodenal switch,” “Endoscopic sleeve gastroplasty,” “Gastric Balloon,” “Gastric bypass surgery,” “mini-gastric bypass,” and “Sleeve gastrectomy.” We did not find a bariatric or alternative surgical technique perceived as not recommended and/or forbidden. Importantly, “mini-gastric bypass” is not officialy recognized by the International Federation for the Surgery of Obesity and Metabolic Disorders (IFSO) [15]. Instead, the IFSO endorses the term “one anastomosis gastric bypass.” However, “mini-gastric bypass” is the name of the topic matching “one anastomosis gastric bypass” in Google Trends. For this reason, search statistics for “mini-gastric bypass” represent queries related to one anastomosis gastric bypass.

We performed data collection on 22nd January 2023. Initially, we typed each topic alone, set the region to “Worldwide,” and generated time trends for 2004–2022 and 2020–2022. That data generated by typing only one topic at once is later called “non-adjusted data.” Further, we analyzed all time trends visually and chose the topic “Gastric bypass surgery” as a benchmark because it has stable RSV during the analyzed timeline, and the topic was one of the most popular. We generated time trends for all topics and “Gastric bypass surgery” (“adjusted data”) for the periods 2004–2022 and 2020–2022, as well as interest broken by region for 2004–2022. Each time we generated adjusted data, we typed the topic “Gastric bypass surgery” with one of the analyzed topic; thus, we collected time trends and geographical data for two topics at once. Consequently, all topics were standardized to a common benchmark for consistency and comparability. All details about search input are reported in Supplementary Table 1 according to the Nuti protocol [16].

### Data Analysis

The data processing protocol was inspired by previous reports utilizing GT data [8, 9]. The data is stored on the Mendeley Data repository [17]. All details about data processing, and statistical analysis are presented in Supplementary File 2.

We utilized Grammarly and ChatGPT to correct spelling and grammar mistakes in our manuscript.

## Results

In 2004–2022, Google users globally were most interested in topics representing recommended surgical methods of weight loss (0.52 ± 0.63, expressed as a ratio with the value of the topic “Gastric bypass surgery”), followed by not recommeded pharmacological methods (0.48 ± 0.68), and recommended pharmacological methods (0.46 ± 0.60) (Table 1). However, in 2020–2022, the recommended pharmacological methods (0.65 ± 0.70) gained popularity over other methods (recommended surgical methods: 0.63 ± 0.82; not recommended pharmacological methods: 0.44 ± 0.78). However, the Kruskal–Wallis test did not show a significant difference between analyzed methods both in 2004–2022 [*H*(2) = 0.50; *p* = 0.78] and in 2020–2022 [*H*(2) = 2.93; *p* = 0.23]; thus, the post hoc comparison was not performed.

**Table 1:** The popularity of categories of topics representing obesity treatment methods in proportion to the benchmark: “Gastric bypass surgery” (adjusted data; relative search volume (RSV) over time)

No	Category of topics	Number of topics	Ratio of RSV to “Gastric bypass surgery” 2004–2022	Ratio of RSV to “Gastric bypass surgery” 2020–2022
1	Recommended surgical methods	9	0.52 ± 0.63	0.63 ± 0.82
2	Not recommended pharmacological methods	34	0.48 ± 0.68	0.44 ± 0.78
3	Recommended pharmacological methods	10	0.46 ± 0.60	0.65 ± 0.70

*RSV* relative search volume

Among all of the analyzed topics, “Flaxseed” followed by “Spirulina,” “Carnitine,” “Bariatric surgery,” and “Orlistat” gained the most popularity in the years 2004–2022 (Table 2). During the COVID-19 pandemic (2020–2022), “Spirulina” became the most popular, followed by “Flaxseed,” “Bariatric surgery,” “Semaglutide,” and “Carnitine.” Across the globe, “Flaxseed” was the most popular of the analyzed topics in *n* = 16 countries, followed by “Spirulina” (*n* = 9), “Carnitine” and “Mangosteen” (*n* = 6), and “Garcinia cambogia” (*n* = 5) (Supplementary Fig. 1). The top ten topics representing analyzed obesity treatment methods in each country are presented in Supplementary Table 2. Among recommended methods, “Orlistat” was the most popular topic in the UK, while “Bariatric surgery” in Brazil and the USA, “Gastric balloon” in Guinea, and “Gastric bypass surgery” in Belgium, Denmark, and Sweden. However, 198 of 250 countries revealed low search volume.

**Table 2:** The popularity of the topics representing weight loss methods in the years 2004–2022 and 2020–2022, as a proportion to the benchmark: “Gastric bypass surgery” (adjusted data; relative search volume (RSV) over time)

No	Topic (2004–2022)	Proportion of RSV to the benchmark	Topic (2020–2022)	Proportion of RSV to the benchmark
1	Flaxseed	2.41	Spirulina	3.08
2	Spirulina	2.10	Flaxseed	2.91
3	Carnitine	2.04	**Bariatric surgery**	**2.40**
**4**	**Bariatric surgery**	**1.84**	*Semaglutide*	2.17
5	*Orlistat*	1.83	Carnitine	1.97
6	Garcinia cambogia	1.70	**Sleeve gastrectomy**	**1.37**
7	*Phentermine*	1.24	*Orlistat*	1.23
8	Sibutramine	1.14	Curcumin	1.18
9	Conjugated linoleic acid	1.05	Mangosteen	1.04
**10**	**Gastric bypass surgery**	**1.00**	*Saxenda*	1.01
11	Mangosteen	0.96	**Gastric bypass surgery**	**1.00**
12	Clenbuterol	0.84	*Phentermine*	0.98
13	**Adjustable gastric band**	0.82	Alpha lipoic acid	0.75
14	**Sleeve gastrectomy**	0.66	Sibutramine	0.67
15	Curcumin	0.64	Conjugated linoleic acid	0.61
16	Alpha lipoic acid	0.58	*Liraglutide*	0.60
17	Kalahari cactus	0.54	Clenbuterol	0.55
18	Chitosan	0.53	Garcinia cambogia	0.50
19	Ephedra	0.40	**Gastric balloon**	0.44
20	*Semaglutide*	0.39	Chitosan	0.37
21	*Liraglutide*	0.38	**Adjustable gastric band**	0.36
22	**Gastric balloon**	0.30	Beta-glucan	0.31
23	*Saxenda*	0.24	*Bupropion* + *naltrexone*	0.28
24	Rimonabant	0.23	Glucomannan	0.21
25	*Amfepramone*	0.21	Bumetanide	0.21
26	Beta-glucan	0.19	Candyleaf	0.20
27	Glucomannan	0.17	Phenylpropanolamine	0.11
28	Phenylpropanolamine	0.17	*Amfepramone*	0.11
29	Candyleaf	0.14	Ephedra	0.10
30	Bumetanide	0.14	*Phentermine* + *topiramate*	0.09
31	*Bupropion* + *naltrexone*	0.13	2,4-Dinitrophenol	0.07
32	Caralluma	0.11	Lorcaserin	0.07
33	Lorcaserin	0.11	**Duodenal switch**	0.04
34	*Phentermine* + *topiramate*	0.08	Kalahari cactus	0.04
35	2,4-Dinitrophenol	0.07	Caralluma	0.03
36	Fenproporex	0.06	*Xenical*	0.03
37	**Duodenal switch**	0.03	Fenproporex	0.03
38	*Benzphetamine*	0.03	**Endoscopic sleeve gastroplasty**	0.03
39	*Xenical*	0.03	**mini-gastric bypass**	0.02
40	Fenfluramine	0.02	Fenfluramine	0.02
41	**Mini-gastric bypass**	0.02	Rimonabant	0.01
42	Dexfenfluramine	0.01	*Benzphetamine*	0.01
43	**Endoscopic sleeve gastroplasty**	0.01	Higenamine	0.01
44	Oxilofrine	0.01	Dexfenfluramine	0.01
45	Cetilistat	0.01	**Biliopancreatic diversion**	0.01
46	Higenamine	0.01	Cetilistat	0.01
47	Vachellia rigidula	0.01	White kidney bean	0.01
48	ß-Methylphenethylamine	0.01	Octodrine	0.01
49	White kidney bean	0.01	ß-Methylphenethylamine	0.01
50	**Biliopancreatic diversion**	0.01	1,3-Dimethylbutylamine	0.01
51	1,3-Dimethylbutylamine	0.01	Vachellia rigidula	0.01
52	Octodrine	0.01	Oxilofrine	0.01
53	Deterenol	0.00	Deterenol	0.01

Topics representing recommended surgical methods are in bold, while those representing recommended pharmacological methods are expressed in italics

*RSV* relative search volume

**Fig. 1:**
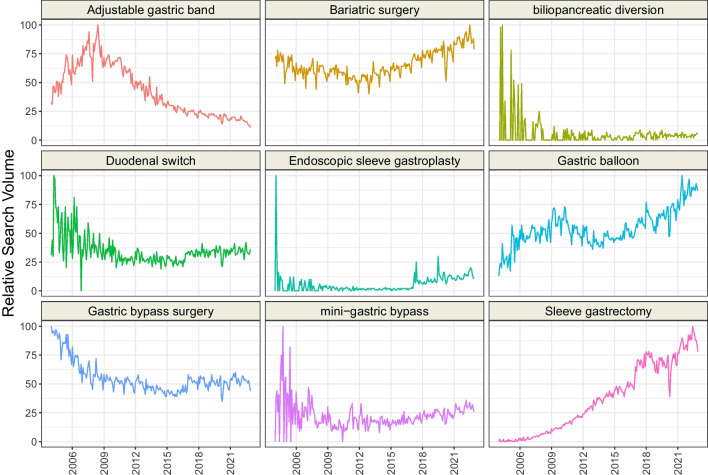
Time trends of relative search volumes of topics representing recommended surgical obesity methods

**Fig. 2:**
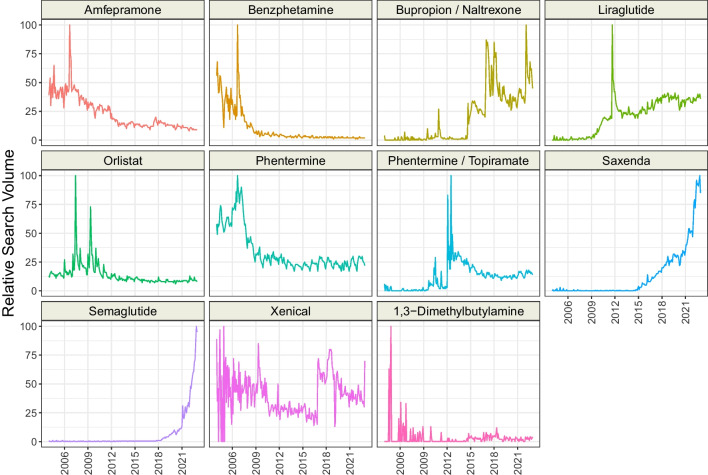
Time trends of relative search volumes of topics representing recommended pharmacological obesity methods

**Fig. 3:**
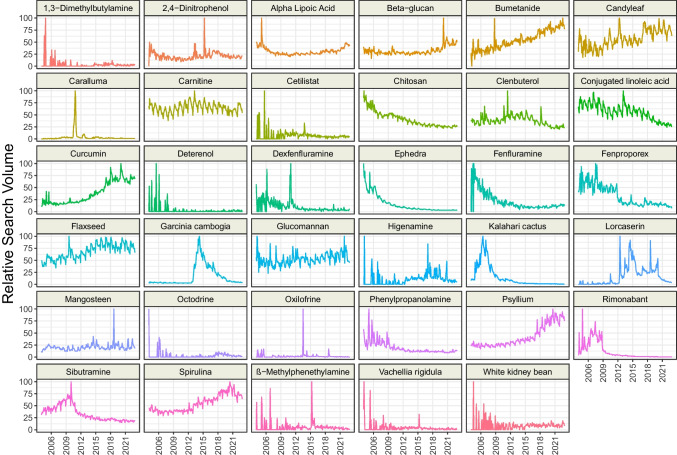
Time trends of relative search volumes of topics representing not recommended pharmacological obesity methods

We visualized the RSV over time of each analyzed topic and presented it in Figs. 1, 2, and 3. Overall, the interest in four topics representing recommended surgical methods has increased since 2004, whereas for four topics it decreased (Table 3). Among recommended pharmacological methods, the interest of Google users in five topics grows, and for another four, it falls. Interest in not recommended pharmacological treatment increased for twelve topics, while thirteen decreased. The most dynamic increase of interest in 2004–2022 was observed for “Sleeve gastrectomy” (+ 5.23 RSV/year), “Curcumin” (+ 3.69 RSV/year), “Psyllium” (+ 3.54 RSV/year), and “Bupropion/Naltrexone” (+ 3.33 RSV/year). The RSV most rapidly decreased for “Adjustable gastric band” (− 3.33 RSV/year), “Fenproporex” (− 3.32 RSV/year), and “Chitosan” (− 2.62 RSV/year). We identified several unusual peaks of interest in the topics: “Amfepramone” (September 2006), “Beznphetamine” (September 2006), “Liraglutide” (September 2011), “Orlistat” (June 2007), “Phentermine / Topiramate” (July 2012), “2,4 − Dinitrophenol” (April 2015), “Alpha Lipoic Acid” (February 2005), “Beta − glucan” (March 2020), “Caralluma” (October–December 2011), “Dexfenfluramine” (January 2011), “Mangosteen” (September 2009), and “Oxilofrine” (July 2013). Moreover, we found that “Saxenda” and “Semaglutide” revealed an almost exponential increase in RSV since 2018.

**Table 3:** Time series analysis of non-adjusted topics

Topic	Category	Seasonal Mann–Kendall test	Slope [RSV/year]
Adjustable gastric band	Approved surgical methods	tau = − 0.70; ***	− 3.33; ***
Bariatric surgery	Approved surgical methods	tau = 0.20; ***	1.22; ***
biliopancreatic diversion	Approved surgical methods	tau = 0.16; **	− 0.51; ***
Duodenal switch	Approved surgical methods	tau = − 0.28; ***	− 0.86; ***
Endoscopic sleeve gastroplasty	Approved surgical methods	tau = 0.27; ***	0.39; ***
Gastric balloon	Approved surgical methods	tau = 0.36; ***	2.00; ***
Gastric bypass surgery	Approved surgical methods	tau = − 0.58; ***	− 1.48; ***
Mini-gastric bypass	Approved surgical methods	tau = 0; 0.98	–
Sleeve gastrectomy	Approved surgical methods	tau = 0.97; ***	5.23; ***
Amfepramone	Approved pharmacological methods	tau = − 0.76; ***	− 2.19; ***
Benzphetamine	Approved pharmacological methods	tau = − 0.84; ***	− 1.88; ***
Bupropion + naltrexone	Approved pharmacological methods	tau = 0.66; ***	3.33; ***
Liraglutide	Approved pharmacological methods	tau = 0.83; ***	2.35; ***
Orlistat	Approved pharmacological methods	tau = − 0.66; ***	− 0.86; ***
Phentermine	Approved pharmacological methods	tau = − 0.67; ***	− 2.39; ***
Phentermine + topiramate	Approved pharmacological methods	tau = 0.43; ***	0.91; ***
Saxenda	Approved pharmacological methods	tau = 0.66; ***	3.03; ***
Semaglutide	Approved pharmacological methods	tau = 0.61; ***	1.59; ***
Xenical	Approved pharmacological methods	tau = − 0.11; 0.035	− 0.06; 0.77
1,3-Dimethylbutylamine	Unapproved pharmacological methods	tau = 0.29; ***	− 0.28; 0.013
2,4-Dinitrophenol	Unapproved pharmacological methods	tau = 0.16; **	0.08; 0.46
Alpha lipoic acid	Unapproved pharmacological methods	tau = − 0.06; 0.24	–
Beta-glucan	Unapproved pharmacological methods	tau = 0.07; 0.22	–
Bumetanide	Unapproved pharmacological methods	tau = 0.77; ***	3.15; ***
Candyleaf	Unapproved pharmacological methods	tau = 0.53; ***	2.36; ***
Caralluma	Unapproved pharmacological methods	tau = 0.1; 0.094	–
Carnitine	Unapproved pharmacological methods	tau = 0.2; ***	0.08; 0.48
Cetilistat	Unapproved pharmacological methods	tau = 0.03; 0.61	− 0.32; **
Chitosan	Unapproved pharmacological methods	tau = − 0.92; ***	− 2.62; ***
Clenbuterol	Unapproved pharmacological methods	tau = 0.01; 0.83	− 0.67; ***
Conjugated linoleic acid	Unapproved pharmacological methods	tau = − 0.59; ***	− 2.41; ***
Curcumin	Unapproved pharmacological methods	tau = 0.8; ***	3.69; ***
Deterenol	Unapproved pharmacological methods	tau = 0.02; 0.79	− 0.46; ***
Dexfenfluramine	Unapproved pharmacological methods	tau = − 0.54; ***	− 1.21; ***
Ephedra	Unapproved pharmacological methods	tau = − 0.97; ***	− 2.60; ***
Fenfluramine	Unapproved pharmacological methods	tau = − 0.75; ***	− 2.48; ***
Fenproporex	Unapproved pharmacological methods	tau = − 0.7; ***	− 3.32; ***
Flaxseed	Unapproved pharmacological methods	tau = 0.7; ***	2.19; ***
Garcinia cambogia	Unapproved pharmacological methods	tau = 0.31; ***	0.89; ***
Glucomannan	Unapproved pharmacological methods	tau = 0.36; ***	0.80; ***
Higenamine	Unapproved pharmacological methods	tau = 0.56; ***	0.60; ***
Kalahari cactus	Unapproved pharmacological methods	tau = − 0.85; ***	− 2.68; ***
Lorcaserin	Unapproved pharmacological methods	tau = 0.66; ***	1.47; ***
Mangosteen	Unapproved pharmacological methods	tau = 0.21; ***	0.33; ***
Octodrine	Unapproved pharmacological methods	tau = 0.36; ***	− 0.09; 0.38
Oxilofrine	Unapproved pharmacological methods	tau = 0.18; **	− 0.07; 0.40
Phenylpropanolamine	Unapproved pharmacological methods	tau = − 0.78; ***	− 1.65; ***
Psyllium	Unapproved pharmacological methods	tau = 0.83; ***	3.54; ***
Rimonabant	Unapproved pharmacological methods	tau = − 0.83; ***	− 2.07; ***
Sibutramine	Unapproved pharmacological methods	tau = − 0.62; ***	− 2.20; ***
Spirulina	Unapproved pharmacological methods	tau = 0.7; ***	2.72; ***
ß-Methylphenethylamine	Unapproved pharmacological methods	tau = 0.31; ***	− 0.17; 0.23
Vachellia rigidula	Unapproved pharmacological methods	tau = 0.01; 0.87	–
White kidney bean	Unapproved pharmacological methods	tau = 0.25; ***	0.04; 0.75

**p < 0.01; ***p < 0.001; *RSV* relative search volume

## Discussion

We examined over 50 GT topics related to various pharmacological and surgical obesity treatments, ranking the most prevalent methods worldwide and studying their temporal trends.

### Main Findings

Bariatric surgery is the most definite, radical weight loss method and the most effective [18]. Over the last two decades, the number of bariatric procedures has increased [19], and this trend is expected to continue in the coming years [20]. Therefore, surgical methods were numerically more popular among Google users than others analyzed categories of obesity treatment methods. Between 2020 and 2022, recommended drugs surpassed numerically in popularity other methods. We assume that the advent of the wide use of GLP-1 analogs caused increased interest in these drugs among Google users. In the pre-GLP-1 analogs era, the use of recommended drugs for obesity was limited due to burdensome side effects (e.g., fatty diarrhea after orlistat intake) or modest efficacy (e.g., phentermine and topiramate). However, Kruskal–Wallis tests did not show significant differences between analyzed categories. We assume that a low number of topics in recommended methods—nine for surgical and ten for pharmacological—constrained the test’s statistical power.

Among all topics, “Sleeve gastrectomy” had the most dynamic increase in RSV over time. “Adjustable gastric band” peaked between 2005 and 2010, and “Gastric bypass surgery” in 2004. Abraham et al. reported a shift from laparoscopic adjustable gastric bands and Roux en Y gastric bypass to sleeve gastrectomy since 2010 [21], which confirms our observations. Moreover, recent advances in endoscopic bariatric procedures may explain the dynamic increase of RSV of “Gastric balloon” [22]. The topics with very low popularity compared to the benchmark (e.g., “Duodenal switch”) often present irregular trends, and it is often hard to explain the causes of such patterns.

The drugs recommended for treating obesity in recent years (semaglutide, liraglutide, bupropion with naltrexone) showed a dynamic increase over time, which may suggest further interest increases among Google users. FDA approved phentermine, amfepramone, and benzphetamine in the ‘50 s, orlistat in 1999, and a combination of phentermine and topiramate in 2012 [23]. The recommended old drugs tended to decrease popularity over time and became a niche, while orlistat and phentermine (alone or with topiramate) had stable interest over time.

The RSV trends of banned anti-obesity drugs associate with their withdrawal. Fenfluramine and dexfenfluramine (in combination with phentermine or not) were withdrawn from the USA in 1997 due to their association with heart valve disease [24], which explains the decrease in RSV over time. FDA approved lorcaserin, but in 2020 the drug was withdrawn due to the increased risk of pancreatic, colorectal, and lung cancers [25]. The interest of Google users in lorcaserin reflected both the approval and withdrawal of the drug by the FDA. RSV of phenylpropanolamine decreased over time due to FDA public health advisory in 2000 [26] and further withdrawal of the drug in many countries. The SCOUT study revealed in 2010 the high risk of sibutramine which led to the withdrawal of the drug from Europe and the USA [27]. GT revealed a peak in sibutramine interest in 2010, followed by a decline, although it remains notably popular due to its availability in certain countries, ranking as the 7th most searched topic from 2004–2022. The interest in rimonabant sharply decreased after 2008, when it was banned in Europe due to serious psychiatric effects [28].

A prior study indicated that certain diets might be faddish, as seen with the Atkins and 5:2 diets [8]. Our observations align, with similar spikes in search interest for dietary supplement ingredients like chitosan, ephedra, kalahari cactus, and garcinia cambogia. This interest may stem from initial promising research, aggressive marketing by manufacturers, and media hype, which wanes when the products fail to deliver expected results.

The peak of interest in “Orlistat” in June 2007 could be attributed to its approval by the FDA and EMA, and the introduction of the first over-the-counter orlistat called Alli® [29, 30]. The peak of the search on “Phentermine/Topiramate” corresponds with FDA approval of the combination of phentermine and topiramate [31]. In July 2013, two top sprinters were tested positive for oxilofrine, which gained media attention [32]. However, we could not identify events that surged the interest of Google users in the other topics.

### Strengths and Practical Implications

This is the very first study that compared the popularity of different surgical and pharmacological methods for treating obesity among Google users across the world. Many of our observations were consistent with clinical practice and contemporary literature, and we may assume that GT may mirror some trends in obesity treatment.

Our study examined a wide range of these interventions for obesity. While professionals prioritize methods backed by research, such as bariatric surgery and new drugs, patients’ preferences can vary. Some opt for the immediate results of surgery, possibly supplemented by medication, while others, cautious of surgical risks, may choose drug treatments. Financial constraints or insurance coverage may also lead some to prefer less expensive or alternative treatments, often perceived as natural and safer than conventional drugs or surgeries.

Research-supported obesity treatments, recognized by both therapists and patients, are likely to rise in popularity, a trend observable through Google Trends. This tool provides insights into treatment preferences and helps professionals recognize the escalating demand for accessible treatments. Google Trends’ ease of use enables comparison between mainstream and alternative obesity treatments and can be applied to analyze the uptake of new, validated therapies like tirzepatide, as well as emerging alternatives, on a global and local scale.

Therapists typically guide patients toward optimal treatment choices on an individual level, while the Internet serves as a broader resource for obesity treatment. The quality and clarity of online information, both globally and locally, are crucial. Yet, the quality of many obesity [33] and bariatric surgery websites is disappointing [34, 35]. It is essential for the professional obesity community to enhance online resources, helping patients to make informed choices that adhere to best practice standards. Additionally, medical professionals should direct patients to trustworthy online sources.

### Limitations

The study has limitations. Firstly, including reliance on Google Trends’ topic matching, which led to the exclusion of certain procedures or brand names like Wegovy®. Secondly, we omitted dietary supplements not primarily linked to weight loss, such as resveratrol, to avoid skewed results from searches for their other known benefits. Drugs prescribed off-label for weight loss, like fluoxetine, were also excluded, affecting the comparison across treatment categories. Thirdly, the categorization of some anti-obesity drugs as recommended is debatable due to changes in their market status since 2004, including market withdrawal of several drugs. While drugs like sibutramine remain available in various countries, amfepramone, though FDA-approved for short-term use, was recently banned in the European Union [36]. Finally, the absence of demographic data (e.g., age and sex) on GT users restricts the analysis of results, and Google’s uneven market share affects user engagement, with notably less in regions such as Russia and the Far East compared to Western countries [37].

## Conclusion

Google users across the world were the most interested in topics representing bariatric surgery, but recently recommended drugs for the treatment of obesity gained the most attention. The most popular individual topics were dietary supplements with uncertain effects on weight loss.

### Supplementary Information

Below is the link to the electronic supplementary material.

Supplementary file1 (PDF 494 KB)

Supplementary file2 (DOC 29 KB)

Supplementary file3 (DOC 15 KB)

Supplementary file4 (DOC 20 KB)

Supplementary file5 (DOC 46 KB)
