# Supplementary material for: Popularity of Surgical and Pharmacological Obesity Treatment Methods Searched by Google Users: the Retrospective Analysis of Google Trends Statistics in 2004–2022
Source: Obes Surg. 2023 Dec 16;34(3):882–91. doi: 10.1007/s11695-023-06971-y (PMC10899289; doi:10.1007/s11695-023-06971-y)
Supplement: Supplementary file 1 — Supplementary file1 (PDF 494 KB) [file 11695_2023_6971_MOESM1_ESM.pdf]

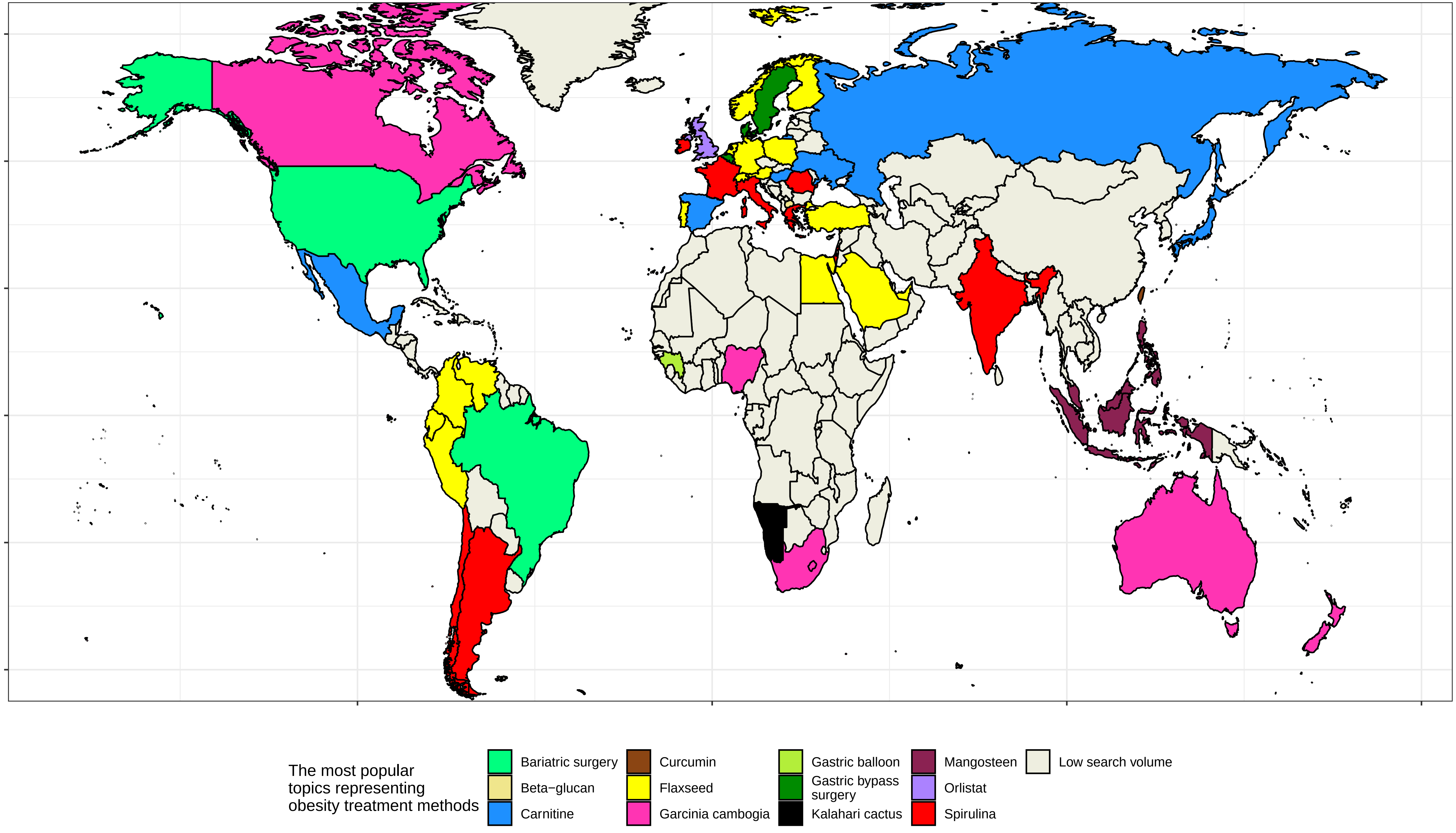
The most popular topics representing obesity treatment methods
Bariatric surgery
Beta−glucan
Carnitine
Curcumin
Flaxseed
Garcinia cambogia
Gastric balloon
Gastric bypass surgery
Kalahari cactus
Mangosteen
Orlistat
Spirulina
Low search volume
