# Supplementary material for: Popularity of Surgical and Pharmacological Obesity Treatment Methods Searched by Google Users: the Retrospective Analysis of Google Trends Statistics in 2004–2022
Source: Obes Surg. 2023 Dec 16;34(3):882–91. doi: 10.1007/s11695-023-06971-y (PMC10899289; doi:10.1007/s11695-023-06971-y)
Supplement: Supplementary file 2 — Supplementary file2 (DOC 29 KB) [file 11695_2023_6971_MOESM2_ESM.doc]

Supplementary File 1

Selection of search terms, and matching with topics.

Firstly, we searched for pharmacological and surgical obesity treatment methods, both recommended and not recommended. We used the PubMed search engine to search for reviews and original articles reporting obesity treatment methods. Additionally, we read U.S. Food and Drug Administration (FDA) [1] and National Institutes of Health (NIH) resources about obesity treatment [2]. We excluded pharmacological agents that are not used mainly for weight loss (e.g., fluoxetine, amphetamine). Moreover, we did not include drugs registered for genetic obesity (e.g., setmelanotide). After the initial selection of obesity treatment methods by M.K. and M.M-D., all included methods were revised and recommended by senior authors (M.K-N. and P.B.). For pharmacological methods, we included the names of the original drugs.

The recommended pharmacological methods for obesity were: orlistat (Xenical®), naltrexone-bupropion (Comcave®, Mysimba®), liraglutide (Saxenda®), semaglutide (Wegovy®), phentermine (Sentis®, Ionamin®), phentermine-topiramate (Qsymia®), benzphetamine (Didrex®), diethylpropion (amfepramone) [2–4]. We matched the following topics (n = 10) representing recommended pharmacological methods of obesity treatment: "Amfenaprone" (matched by typing "diethylpropion"), "Benzphetamine", "Bupropion / Naltrexone", "Liraglutide", "Orlistat", "Phentermine", "Phentermine / Topiramate", "Saxenda", "Semaglutide", and "Xenical".

We selected following unapproved and/or not recommended pharmacological (mostly dietary supplements) method for treatment of obesity: β-Glucans [5], 1,3-dimethylamylamine [6], 1,3-dimethylbutylamine [7], 1,4-dimethylamylamine [7], 2,4-dinitrophenol [8–10], Acacia (Vachellia) rigidula [10], aminorex (Aminocel®) [11], beta-methyl-phenylethylamine [6], bitter orange [5], bumetanide (Bumex®) [12,13], Camellia sinensis [10], caralluma [5], carnitine [10], cetilistat [1], chitosan [10], Citrus aurantium [14], clenbuterol [15,16], conjugated linoleic acid [10], curcumin [5], deterenol [7], dexfenfluramine [10], diacylglycerol [5], ephedra [6], fenfluramine (Fintepla®) [10], fenproporex (Perphoxene®) [17], flaxseed [5], garcinia cambogia [18], glucomannan [10], guar gum [5], higenamine [7], hoodia gordonii [10], licorice [5], lipoic acid [5], lorcaserin (Belviq®) [11], mangosteen [5], octodrine [7], oxilofrine [7], phenpromethamine [7], phenylpropanolamine [10], rimonabant (Acomplia®) [10], sibutramine (Meridia®, Reductil®) [19], spirulina [5], Stevia rebaudiana (candyleaf) [10], white kidney bean [5]. We matched the following topics (n = 34) in the GT search engine: "β-Methylphenethylamine", "1,3-Dimethylbutylamine", "2,4-Dinitrophenol", "Alpha Lipoic Acid", "Beta-glucan", "Bumetanide", "Candyleaf", "Caralluma", "Carnitine", "Cetilistat", "Chitosan", "Clenbuterol", "Conjugated linoleic acid", "Curcumin", "Deterenol", "Dexfenfluramine", "Ephedra", "Fenfluramine", "Fenproporex", "Flaxseed", "Garcinia Cambogia", "Glucomannan", "Higenamine", "Kalahari cactus" (matched hoodia gordonii), "Lorcaserin", "Mangosteen", "Octodrine", "Oxilofrine", "Phenylpropanolamine", "Rimonabant", "Sibutramine", "Spirulina", "Vachellia rigidula", and "White kidney bean".

The recommended surgical methods were: "bariatric surgery", a) endoscopic techniques: "intragastric balloon", "endoscopic sleeve", "endoluminal bypass"; b) restrictive surgical techniques: "adjustable gastric band", "vertical gastrectomy" or "gastric sleeve"; c) mixed surgical techniques: "Roux-en-Y gastric bypass", "mini gastric bypass" or "one gastric bypass anastomosis", "biliopancreatic diversion", "gastroileal bypass", "duodenal switch", "single anastomosis duodenoileal bypass with sleeve gastrectomy" [20]. We matched the following topics (n = 9): "Adjustable gastric band", "Bariatric surgery", "biliopancreatic diversion", "Duodenal switch", "Endoscopic sleeve gastroplasty", "Gastric Balloon", "Gastric bypass surgery", "mini-gastric bypass", and "Sleeve gastrectomy". Importantly, we included the topic "Bariatric surgery" to not limit topics representing only specific surgical methods but also the topic representing the whole branch of surgery. We did not find a bariatric or alternative surgical technique perceived as not recommended and/or forbidden.

References

1. Questions and Answers about FDA’s Initiative Against Contaminated Weight Loss Products [Internet]. 2021 [cited 2023 Jan 5]. Available from: https://www.fda.gov/drugs/frequently-asked-questions-popular-topics/questions-and-answers-about-fdas-initiative-against-contaminated-weight-loss-products

2. Prescription Medications to Treat Overweight & Obesity [Internet]. National Institute of Diabetes and Digestive and Kidney Diseases; 2021 [cited 2022 Nov 14]. Available from: https://www.niddk.nih.gov/health-information/weight-management/prescription-medications-treat-overweight-obesity

3. Gomez G, Stanford FC. US health policy and prescription drug coverage of FDA-approved medications for the treatment of obesity. Int J Obes (Lond). 2018;42:495–500.

4. Ferrulli A, Terruzzi I, Senesi P, Succi M, Cannavaro D, Luzi L. Turning the clock forward: New pharmacological and non pharmacological targets for the treatment of obesity. Nutrition, Metabolism and Cardiovascular Diseases. 2022;32:1320–34.

5. Watanabe M, Risi R, Masi D, Caputi A, Balena A, Rossini G, et al. Current Evidence to Propose Different Food Supplements for Weight Loss: A Comprehensive Review. Nutrients. 2020;12:2873.

6. Eichner S, Maguire M, Shea LA, Fete MG. Banned and discouraged-use ingredients found in weight loss supplements. Journal of the American Pharmacists Association. 2016;56:538–43.

7. Cohen PA, Travis JC, Vanhee C, Ohana D, Venhuis BJ. Nine prohibited stimulants found in sports and weight loss supplements: deterenol, phenpromethamine (Vonedrine), oxilofrine, octodrine, beta-methylphenylethylamine (BMPEA), 1,3-dimethylamylamine (1,3-DMAA), 1,4-dimethylamylamine (1,4-DMAA), 1,3-dimethylbutylamine (1,3-DMBA) and higenamine. Clinical Toxicology. 2021;59:975–81.

8. Freeman N, Moir D, Lowis E, Tam E. 2,4-Dinitrophenol: “diet” drug death following major trauma. Anaesth Rep. 2021;9:106–9.

9. Grundlingh J, Dargan PI, El-Zanfaly M, Wood DM. 2,4-dinitrophenol (DNP): a weight loss agent with significant acute toxicity and risk of death. J Med Toxicol. 2011;7:205–12.

10. Koncz D, Tóth B, Roza O, Csupor D. A Systematic Review of the European Rapid Alert System for Food and Feed: Tendencies in Illegal Food Supplements for Weight Loss. Front Pharmacol. 2021;11:611361.

11. Tak YJ, Lee SY. Long-Term Efficacy and Safety of Anti-Obesity Treatment: Where Do We Stand? Curr Obes Rep. 2021;10:14–30.

12. Khazan M, Hedayati M, Kobarfard F, Askari S, Azizi F. Identification and determination of synthetic pharmaceuticals as adulterants in eight common herbal weight loss supplements. Iran Red Crescent Med J. 2014;16:e15344.

13. Zeng Y, Xu Y, Kee C-L, Low M-Y, Ge X. Analysis of 40 weight loss compounds adulterated in health supplements by liquid chromatography quadrupole linear ion trap mass spectrometry. Drug Test Anal. 2016;8:351–6.

14. Stohs SJ. Safety, Efficacy, and Mechanistic Studies Regarding Citrus aurantium (Bitter Orange) Extract and p-Synephrine. Phytother Res. 2017;31:1463–74.

15. Lopes PM, Albuquerque F, Ferreira AM, Trabulo M. Clenbuterol-induced myocarditis in a young man desiring to lose weight. BMJ Case Rep. 2022;15:e247898.

16. Spiller HA, James KJ, Scholzen S, Borys DJ. A Descriptive Study of Adverse Events from Clenbuterol Misuse and Abuse for Weight Loss and Bodybuilding. Substance Abuse. 2013;34:306–12.

17. Garcia Ramirez AV, Filho DR, Zotarelli Filho IJ. Meta-analysis and Approach of the Real Impact of Anorexigenic Drugs in the Obesity in Humans: The Last Five Years of the Randomized Studies. Curr Diabetes Rev. 2020;16:750–8.

18. McCarthy RE, Bowen DG, Strasser SI, McKenzie C. The dangers of herbal weight loss supplements: a case report of drug-induced liver injury secondary to Garcinia cambogia ingestion. Pathology. 2021;53:545–7.

19. Jairoun AA, Al-Hemyari SS, Shahwan M, Zyoud SH. Adulteration of Weight Loss Supplements by the Illegal Addition of Synthetic Pharmaceuticals. Molecules. 2021;26:6903.

20. Luesma MJ, Fernando J, Cantarero I, Lucea P, Santander S. Surgical Treatment of Obesity. Special Mention to Roux-en-Y Gastric Bypass and Vertical Gastrectomy. Front Endocrinol. 2022;13:867838.
