# Supplementary material for: Popularity of Surgical and Pharmacological Obesity Treatment Methods Searched by Google Users: the Retrospective Analysis of Google Trends Statistics in 2004–2022
Source: Obes Surg. 2023 Dec 16;34(3):882–91. doi: 10.1007/s11695-023-06971-y (PMC10899289; doi:10.1007/s11695-023-06971-y)
Supplement: Supplementary file 3 — Supplementary file3 (DOC 15 KB) [file 11695_2023_6971_MOESM3_ESM.doc]

Supplementary File 2

Data processing and statistical analysis

Data manipulation, calculation, and visualization were performed using R 3.6.1 (R Foundation, Vienna, Austria). Firstly, we transformed all RSVs reported by GT as "0" to "0.1" and "<1%" to "0.5".

We used the adjusted data to calculate the mean ratio of the adjusted RSV to "Gastric bypass surgery" for each topic in 2004-2022 and 2020-2022. The topic "Gastric bypass surgery" was represented by a value of 1.00. Furthermore, we calculated a mean adjusted RSV for each of three categories of topics: 1) recommended surgical methods, 2) recommended pharmacological methods, and 3) not recommended pharmacological methods. Finally, we performed the Kruskal-Wallis test to compare if the three analyzed categories differ significantly. We aimed to perform a post-hoc Mann-Whitney U test if the p-value of the Kruskal-Wallis test would be significant (p < 0.05).

To calculate which topic was the most popular in each country in 2004-2022, we used the adjusted data broken down by region, which represents the ratio between the RSV of a topic and
"Gastric bypass surgery" in a specific region. For the option "Worldwide", GT chooses countries as the regions. The sum of both RSVs in any given area adds to 100 (in our case, the benchmark: "Gastric bypass surgery" and another topic). That allows a comparison of which searches related to the topics are more often searched in a given region (country). We calculated the most frequent topics representing surgical or pharmacological obesity treatment methods for all countries with a significant search volume. Because the popularity of the topics was always adjusted to the topic "Gastric bypass surgery", we set the RSV of "Gastric bypass surgery" in a given region to 50. Using that approach, we could have enlisted the topics more or less popular than the benchmark in each country. We identified up to ten most popular topics in each country.

We used generated time trends (the unadjusted data) for time series analysis. Firstly, the seasonal Mann–Kendall was performed to identify a significant (p-value below 0.05) secular trend in each time series [1]. Secondly, we performed a univariate linear regression for all significant secular trends to calculate the slope, expressed as changes in RSV per year in 2004-2022.

References

1. McLeod AI. Kendall rank correlation and Mann-Kendall trend test [Internet]. CRAN; 2011 [cited 2019 Jul 21]. Available from: https://cran.r-project.org/web/packages/Kendall/Kendall.pdf
