# Supplementary material for: Popularity of Surgical and Pharmacological Obesity Treatment Methods Searched by Google Users: the Retrospective Analysis of Google Trends Statistics in 2004–2022
Source: Obes Surg. 2023 Dec 16;34(3):882–91. doi: 10.1007/s11695-023-06971-y (PMC10899289; doi:10.1007/s11695-023-06971-y)
Supplement: Supplementary file 4 — Supplementary file4 (DOC 20 KB) [file 11695_2023_6971_MOESM4_ESM.doc]

Supplementary Table 1.

Checklist for Documentation of Google Trends research. Modified from Nuti et al.

| **Section/Topic** | **Checklist item** |
| --- | --- |
| **Search Variables** |  |
| Access Date | 22 January 2023 |
| Time Periods | a) From January 2004 to the end of 2022  b) From January 2020 to the end of 2022 |
| Query Category | All query categories were used |
| Region | Worldwide |
| Countries with Low Search Volume | Excluded |
| **Search Input** |  |
| **Non-adjusted** | **Recommended pharmacological methods**:  "Amfenaprone", "Benzphetamine", "Bupropion / Naltrexone", "Liraglutide", "Orlistat", "Phentermine", "Phentermine / Topiramate", "Saxenda", "Semaglutide", and "Xenical"  **Recommended surgical methods**:  "Adjustable gastric band", "Bariatric surgery", "biliopancreatic diversion", "Duodenal switch", "Endoscopic sleeve gastroplasty", "Gastric Balloon", "Gastric bypass surgery", "mini-gastric bypass", and "Sleeve gastrectomy"  **Not recommended pharmacological methods**:  "β-Methylphenethylamine", "1,3-Dimethylbutylamine", "2,4-Dinitrophenol", "Alpha Lipoic Acid", "Beta-glucan", "Bumetanide", "Candyleaf", "Caralluma", "Carnitine", "Cetilistat", "Chitosan", "Clenbuterol", "Conjugated linoleic acid", "Curcumin", "Deterenol", "Dexfenfluramine", "Ephedra", "Fenfluramine", "Fenproporex", "Flaxseed", "Garcinia Cambogia", "Glucomannan", "Higenamine", "Kalahari cactus" (matched hoodia gordonii), "Lorcaserin", "Mangosteen", "Octodrine", "Oxilofrine", "Phenylpropanolamine", "Rimonabant", "Sibutramine", "Spirulina", "Vachellia rigidula", and "White kidney bean" |
| **Adjusted** | Topics: "Gastric bypass surgery" + "Amfenaprone" / "Benzphetamine" / "Bupropion / Naltrexone" / "Liraglutide" / "Orlistat" / "Phentermine" / "Phentermine / Topiramate" / "Saxenda" / "Semaglutide" / "Xenical" / "Adjustable gastric band" / "Bariatric surgery" / "biliopancreatic diversion" / "Duodenal switch" / "Endoscopic sleeve gastroplasty" / "Gastric Balloon" / "mini-gastric bypass" / "Sleeve gastrectomy" / "β-Methylphenethylamine" / "1,3-Dimethylbutylamine" / "2,4-Dinitrophenol" / "Alpha Lipoic Acid" / "Beta-glucan" / "Bumetanide" / "Candyleaf" / "Caralluma" / "Carnitine" / "Cetilistat" / "Chitosan" / "Clenbuterol" / "Conjugated linoleic acid" / "Curcumin" / "Deterenol" / "Dexfenfluramine" / "Ephedra" / "Fenfluramine" / "Fenproporex" / "Flaxseed" / "Garcinia Cambogia" / "Glucomannan" / "Higenamine" / "Kalahari cactus" (matched hoodia gordonii) / "Lorcaserin" / "Mangosteen" / "Octodrine" / "Oxilofrine" / "Phenylpropanolamine" / "Rimonabant" / "Sibutramine" / "Spirulina" / "Vachellia rigidula" / "White kidney bean" |
| **Rationale for Search Strategy** |  |
| For Search Input | The searched topics represent different (both recommended and not recommended) methods of weight loss. Because Google Trends enables us to compare only five inputs at once we compared the relative search volume of all topics with the topic "Gastric bypass surgery" (adjusted data). Therefore, we were able to compare all 54 topics in further analysis. |
| For Setting Chosen | We chose all categories to not limit the output. We excluded countries with low search volume, which may be sensitive to outliers. |
