# Supplementary material for: Popularity of Surgical and Pharmacological Obesity Treatment Methods Searched by Google Users: the Retrospective Analysis of Google Trends Statistics in 2004–2022
Source: Obes Surg. 2023 Dec 16;34(3):882–91. doi: 10.1007/s11695-023-06971-y (PMC10899289; doi:10.1007/s11695-023-06971-y)
Supplement: Supplementary file 5 — Supplementary file5 (DOC 46 KB) [file 11695_2023_6971_MOESM5_ESM.doc]

Supplementary Table 2

The ten most common topics representing surgical or pharmacological treatment methods of obesity in a specific country.

Relative search volume was estimated using adjusted data showing the breakdown by region ("Gastric bypass surgery" + another topic).

| Country | The most common topic |
| --- | --- |
| Argentina | Spirulina (73), Flaxseed (68), Alpha Lipoic Acid (64), Conjugated linoleic acid (64), Carnitine (59), Bariatric surgery (57), Garcinia cambogia (50), Gastric bypass surgery (50), Orlistat (48), Clenbuterol (32) |
| Australia | Garcinia cambogia (79), Bariatric surgery (73), Sleeve gastrectomy (72), Clenbuterol (66), Adjustable gastric band (65), Spirulina (65), Semaglutide (62), Carnitine (62), Curcumin (56), Saxenda (50) |
| Austria | Flaxseed (72), Carnitine (55), Spirulina (54), Gastric bypass surgery (50), Orlistat (31), Curcumin (31), Adjustable gastric band (30), Garcinia cambogia (30), Alpha Lipoic Acid (28), Sibutramine (26) |
| Belgium | Gastric bypass surgery (50), Spirulina (41), Flaxseed (38), Gastric balloon (27), Sleeve gastrectomy (26), Carnitine (26), Orlistat (23), Semaglutide (23), Sibutramine (16), Adjustable gastric band (15) |
| Brazil | Bariatric surgery (99), Flaxseed (98), Sibutramine (98), Orlistat (97), Semaglutide (94), Carnitine (94), Spirulina (94), Liraglutide (93), Saxenda (93), Chitosan (88) |
| Brunei | Mangosteen (100), Gastric bypass surgery (50) |
| Canada | Garcinia cambogia (74), Bariatric surgery (66), Flaxseed (66), Semaglutide (65), Spirulina (60), Conjugated linoleic acid (54), Carnitine (50), Gastric bypass surgery (50), Clenbuterol (47), Curcumin (47) |
| Chile | Spirulina (80), Flaxseed (78), Bariatric surgery (63), Phentermine (62), Orlistat (60), Carnitine (60), Sibutramine (57), Gastric bypass surgery (50), Sleeve gastrectomy (49), Conjugated linoleic acid (45) |
| Colombia | Flaxseed (89), Orlistat (81), Spirulina (79), Carnitine (76), Mangosteen (72), Garcinia cambogia (71), Bariatric surgery (66), Sibutramine (63), Conjugated linoleic acid (59), Liraglutide (54) |
| Denmark | Gastric bypass surgery (50), Spirulina (46), Semaglutide (42), Orlistat (41), Liraglutide (35), Saxenda (35), Clenbuterol (34), Flaxseed (31), Carnitine (30), Sleeve gastrectomy (28) |
| Ecuador | Flaxseed (89), Orlistat (76), Spirulina (76), Carnitine (72), Bariatric surgery (65), Sleeve gastrectomy (63), Garcinia cambogia (59), Clenbuterol (53), Conjugated linoleic acid (53), Sibutramine (50) |
| Egypt | Flaxseed (89), Carnitine (84), Orlistat (77), Sleeve gastrectomy (74), Curcumin (66), Spirulina (64), Gastric balloon (50), Gastric bypass surgery (50), Chitosan (46), Bariatric surgery (45) |
| Finland | Flaxseed (86), Spirulina (84), Bariatric surgery (83), Semaglutide (82), Orlistat (81), Clenbuterol (78), Conjugated linoleic acid (78), Carnitine (77), Liraglutide (70), Curcumin (68) |
| France | Spirulina (83), Gastric balloon (67), Flaxseed (66), Sleeve gastrectomy (65), Gastric bypass surgery (50), Adjustable gastric band (44), Carnitine (43), Bariatric surgery (40), Orlistat (40), Conjugated linoleic acid (40) |
| Germany | Flaxseed (81), Carnitine (73), Spirulina (70), Orlistat (54), Gastric bypass surgery (50), Curcumin (49), Alpha Lipoic Acid (44), Adjustable gastric band (43), Sleeve gastrectomy (41), Sibutramine (39) |
| Greece | Spirulina (96), Flaxseed (95), Candyleaf (92), Carnitine (92), Conjugated linoleic acid (89), Clenbuterol (80), Curcumin (79), Orlistat (77), Garcinia cambogia (77), Alpha Lipoic Acid (74) |
| Guinea | Gastric balloon (100), Gastric bypass surgery (50) |
| Hungary | Carnitine (94), Flaxseed (92), Spirulina (92), Kalahari cactus (86), Sibutramine (84), Conjugated linoleic acid (83), Garcinia cambogia (76), Beta-glucan (69), Orlistat (68), Clenbuterol (67) |
| India | Spirulina (91), Flaxseed (90), Garcinia cambogia (90), Carnitine (88), Curcumin (84), Bariatric surgery (82), Mangosteen (77), Clenbuterol (75), Alpha Lipoic Acid (74), Conjugated linoleic acid (73) |
| Indonesia | Mangosteen (100), Spirulina (100), Chitosan (95), Garcinia cambogia (94), Orlistat (93), Curcumin (92), Phenylpropanolamine (88), Carnitine (87), Candyleaf (86), Bariatric surgery (83) |
| Ireland | Spirulina (75), Garcinia cambogia (69), Flaxseed (64), Adjustable gastric band (63), Bariatric surgery (62), Orlistat (62), Carnitine (61), Semaglutide (60), Clenbuterol (59), Conjugated linoleic acid (58) |
| Israel | Spirulina (86), Curcumin (75), Adjustable gastric band (71), Bariatric surgery (69), Liraglutide (66), Flaxseed (58), Carnitine (53), Gastric bypass surgery (50), Phentermine (48), Semaglutide (48) |
| Italy | Spirulina (87), Garcinia cambogia (86), Carnitine (84), Alpha Lipoic Acid (65), Bariatric surgery (64), Sleeve gastrectomy (62), Conjugated linoleic acid (62), Orlistat (57), Candyleaf (55), Glucomannan (50) |
| Japan | Carnitine (96), Spirulina (95), Chitosan (92), Mangosteen (92), Glucomannan (87), Alpha Lipoic Acid (86), Conjugated linoleic acid (85), Candyleaf (84), Curcumin (83), Xenical (80) |
| Macao | Mangosteen (100), Gastric bypass surgery (50) |
| Malaysia | Mangosteen (95), Spirulina (92), Garcinia cambogia (90), Carnitine (78), Bariatric surgery (77), Curcumin (73), Orlistat (71), Chitosan (70), Flaxseed (70), Alpha Lipoic Acid (67) |
| Mexico | Carnitine (90), Flaxseed (87), Orlistat (86), Phentermine (82), Spirulina (82), Clenbuterol (78), Garcinia cambogia (76), Sibutramine (72), Conjugated linoleic acid (71), Sleeve gastrectomy (57) |
| Namibia | Kalahari cactus (100), Gastric bypass surgery (50) |
| Netherlands | Flaxseed (64), Gastric bypass surgery (50), Spirulina (45), Adjustable gastric band (32), Carnitine (31), Garcinia cambogia (25), Curcumin (24), Orlistat (20), Clenbuterol (20), Semaglutide (15) |
| New Zealand | Garcinia cambogia (75), Bariatric surgery (63), Spirulina (63), Gastric bypass surgery (50), Sleeve gastrectomy (49), Carnitine (49), Clenbuterol (43), Flaxseed (43), Adjustable gastric band (39), Curcumin (39) |
| Nigeria | Garcinia cambogia (66), Orlistat (64), Conjugated linoleic acid (55), Gastric bypass surgery (50), Chitosan (48), Curcumin (48), Alpha Lipoic Acid (42), Beta-glucan (17), 2,4-Dinitrophenol (9), Higenamine (1) |
| North Macedonia | Beta-glucan (100), Candyleaf (100), Clenbuterol (100), Glucomannan (100), Spirulina (100), Gastric bypass surgery (50) |
| Norway | Flaxseed (65), Spirulina (58), Phenylpropanolamine (53), Bupropion+Naltrexone (50), Gastric bypass surgery (50), Semaglutide (49), Saxenda (44), Orlistat (42), Sleeve gastrectomy (38), Clenbuterol (38) |
| Peru | Flaxseed (95), Spirulina (92), Carnitine (87), Garcinia cambogia (85), Orlistat (82), Clenbuterol (80), Sleeve gastrectomy (74), Conjugated linoleic acid (72), Adjustable gastric band (70), Bariatric surgery (65) |
| Philippines | Mangosteen (96), Spirulina (95), Garcinia cambogia (93), Carnitine (89), Orlistat (88), Phenylpropanolamine (77), Alpha Lipoic Acid (72), Curcumin (72), Flaxseed (72), Conjugated linoleic acid (69) |
| Poland | Flaxseed (100), Carnitine (98), Spirulina (98), Conjugated linoleic acid (95), Orlistat (94), Semaglutide (94), Clenbuterol (94), Curcumin (93), Alpha Lipoic Acid (92), Sibutramine (92) |
| Portugal | Flaxseed (89), Carnitine (83), Conjugated linoleic acid (83), Spirulina (82), Orlistat (73), Bariatric surgery (70), Mangosteen (70), Garcinia cambogia (63), Sibutramine (62), Clenbuterol (54) |
| Romania | Spirulina (98), Carnitine (95), Flaxseed (95), Curcumin (89), Amfepramone (88), Sibutramine (87), Garcinia cambogia (86), Orlistat (82), Sleeve gastrectomy (77), Chitosan (77) |
| Russia | Carnitine (97), Sibutramine (97), Spirulina (91), Orlistat (89), Alpha Lipoic Acid (88), Clenbuterol (85), Flaxseed (85), Mangosteen (77), Curcumin (74), Chitosan (71) |
| Saudi Arabia | Flaxseed (96), Sleeve gastrectomy (91), Carnitine (81), Gastric balloon (73), Orlistat (70), Spirulina (70), Adjustable gastric band (68), Liraglutide (62), Saxenda (58), Semaglutide (58) |
| Singapore | Mangosteen (92), Spirulina (89), Garcinia cambogia (85), Curcumin (80), Flaxseed (78), Carnitine (75), Conjugated linoleic acid (74), Bariatric surgery (70), Orlistat (67), Clenbuterol (66) |
| South Africa | Garcinia cambogia (86), Clenbuterol (67), Conjugated linoleic acid (65), Spirulina (59), Bariatric surgery (50), Gastric bypass surgery (50), Kalahari cactus (48), Carnitine (47), Flaxseed (43), Curcumin (42) |
| Spain | Carnitine (86), Spirulina (86), Flaxseed (83), Garcinia cambogia (81), Orlistat (77), Gastric balloon (72), Clenbuterol (69), Conjugated linoleic acid (69), Chitosan (63), Bariatric surgery (59) |
| Sweden | Gastric bypass surgery (50), Flaxseed (47), Spirulina (37), Orlistat (25), Sleeve gastrectomy (24), Phenylpropanolamine (24), Sibutramine (23), Carnitine (17), Clenbuterol (17), Garcinia cambogia (17) |
| Switzerland | Flaxseed (58), Spirulina (58), Gastric bypass surgery (50), Carnitine (49), Garcinia cambogia (40), Orlistat (31), Semaglutide (24), Saxenda (23), Mangosteen (23), Gastric balloon (22) |
| Taiwan | Curcumin (95), Mangosteen (92), Carnitine (77), Spirulina (76), Ephedra (65), Flaxseed (64), Chitosan (62), Alpha Lipoic Acid (61), Conjugated linoleic acid (56), Candyleaf (50) |
| Turkey | Flaxseed (95), Carnitine (94), Sleeve gastrectomy (88), Curcumin (84), Spirulina (83), Conjugated linoleic acid (81), Beta-glucan (75), Orlistat (72), Alpha Lipoic Acid (72), Gastric balloon (71) |
| Ukraine | Carnitine (98), Spirulina (97), Sibutramine (95), Flaxseed (93), Orlistat (92), Alpha Lipoic Acid (90), Clenbuterol (90), Chitosan (86), Mangosteen (86), Curcumin (85) |
| United Arab Emirates | Flaxseed (82), Carnitine (77), Garcinia cambogia (73), Spirulina (70), Bariatric surgery (60), Sleeve gastrectomy (60), Conjugated linoleic acid (59), Mangosteen (58), Orlistat (57), Clenbuterol (54) |
| United Kingdom | Orlistat (72), Spirulina (65), Adjustable gastric band (64), Bariatric surgery (62), Clenbuterol (61), Garcinia cambogia (60), Flaxseed (58), Conjugated linoleic acid (56), Carnitine (51), Sleeve gastrectomy (50) |
| United States | Bariatric surgery (66), Garcinia cambogia (64), Phentermine (63), Gastric bypass surgery (50), Sleeve gastrectomy (46), Flaxseed (46), Spirulina (41), Semaglutide (38), Orlistat (37), Conjugated linoleic acid (37) |
| Venezuela | Flaxseed (88), Carnitine (85), Orlistat (74), Bariatric surgery (58), Garcinia cambogia (56), Spirulina (54), Sibutramine (52), Clenbuterol (50), Gastric bypass surgery (50), Conjugated linoleic acid (45) |
